# An Intron Variant of SLC2A9 Increases the Risk for Type 2 Diabetes Mellitus Complicated with Hyperuricemia in Chinese Male Population

**Published:** 2018-06

**Authors:** Xuan-Long YI, Jiang LI, Dong-Mei MENG, Yan-Jun LIU, Yan-Hong LIU, Hong-Min MA, Ying YUAN, Shi-Chao XING

**Affiliations:** 1. The Affiliated Hospital of Qingdao University, Qingdao, Shandong Province, China; 2. The College of Life Science, Nanjing Agricultural University, Nanjing, China; 3. Dept. of Scientific Research Shandong Institute of Orthopaedics and Traumatology, Qingdao, Shandong Province, China

**Keywords:** SLC2A9, Type 2 diabetes mellitus, Hyperuricemia, Pancreatic β cells

## Abstract

**Background:**

The aim of this study was to explore the associations of haplotypes of the glucose transporter 9 (SLC2A9) genes with type 2 diabetes mellitus (T2DM) complicated with hyperuricemia (HUA).

**Methods:**

Overall, 608 Chinese males, enrolled from the Affiliated Hospital of Medical College of Qingdao University in 2009–2012, were genotyped. The subjects included 167 withT2DM (average age of onset (58.07±11.82 yr), 198 with HUA subjects (average age of onset (39.20±9.73) yr), 115 with T2DM complicated with HUA (average age of onset (51.24±10.09) yr), and 128 control subjects (average age (41.92±10.01) yr). Patients genotypes of the SNPs; including rs734553 was determined by PCR method. Each genotype was regressed assuming the co-dominant, dominant and the recessive models of inheritance with covariates of duration of total glucose, uric acid, urea nitrogen, triglyceride, cholesterol, and creatinine levels.

**Results:**

Chi-square test revealed that rs734553polymorphism was both significantly associated with HUA as well as T2DM complicated HUA, but not with pure T2DM. After adjustment for age and gender, analysis showed that people with C allele had higher risk of HUA and T2DM complicated HUA than those without C allele. And none of the subjects had the homozygous genotype for SLC2A9 (CC).

**Conclusion:**

The SLC2A9 mutation increases the risk for T2DM complicated HUA in Chinese population, which suggested that intron variants between two relatively conserved exons could also be associated with diseases. In patients of T2DM complicated with HUA, the diagnosis and detection of SLC2A9 gene variants should be caused enough attention.

## Introduction

The incidence of metabolic syndrome is increasing worldwide; this is especially true for Type 2 Diabetes Mellitus (T2DM) (1) and Hyperuricemia (HUA) (2), which often occurs together. Among Chinese T2DM patients with central obesity, the results of the current study show prevalence of HUA in women as 36.1% and in men as 28.4% (3). T2DM and HUA are caused by mutations in genes. SLC2A9 is also known as glucose transporter9 (GLUT9) or urate efflux transporter (URAT)v1, which is a high-capacity uric acid transporter as well as a glucose transporter(4). Recently, it was identified to be a modulator of uric acid and plays critical roles in maintaining glucose and uric acid homeostasis. SLC2A9 has two variants, SLC2A9v1 usually trafficked to the basolateral membrane of proximal tubule epithelial cells and SLC2A9v2 localized to the apical membrane and has a high capacity for the urate transport (5). The mechanism of uric acid excretion by SLC2A9 regulation is through the two variants (6). Recently, common genetic variants of SLC2A9 be strongly associated with serum urate level and gout in Caucasian cohorts from Italy, UK, Croatia, the United States, Germany, and Austria (7, 8). Furthermore, GLUT9 also regulate insulin secretion in the pancreatic β cell and up-regulated in the diabetic mouse (9). As a dual transporter for fructose and uric acid, it is necessary to investigate whether there are genetic variants of SLC2A9 affect the glucose and uric acid homeostasis in patients.

SLC2A9 has a relatively conserved amino acid sequence in the seventh and eighth helices located around the central channel of the transport protein (10), which makes the variant in intron7 very important. The rs734553 polymorphism is loci in the intron7 of the SLC2A9, reported to influence serum uric acid levels contributing to susceptibility to gout, Parkinson’s disease or chronic kidney disease (CKD) Progression (11–14). Therefore, it is a better candidate variant to investigate the possible association between the glucose and uric acid homeostasis in patients.

The aim of this study was to explore the associations of haplotypes of the SLC2A9 genes with T2DM complicated with HUA.

## Materials and Methods

### Ethics statement

The study has been approved by the ETHICAL COMMITTEE of the Affiliated Hospital of Qingdao University and has been performed in accordance with the ethical standards of the National Research Centre committee and with the 1964 Helsinki Declaration and its later amendments or comparable ethical standards. Informed consent was obtained from all individual participants included in the study.

### Study subjects

The study subjects were people who attended the Affiliated Hospital of Qingdao University Medical School, including its clinics, wards, and medical center, between 2009 and 2012. We identified 608 males aged 30–70 yr to provide a cohort of subjects from the same population living in the coastal area of Shandong Province, China. Impaired glucose tolerance (IGT), impaired fasting glucose (IFG), and HUA were diagnosed by clinical endocrinologists using established criteria. IFG was defined as fasting plasma glucose of 6.1–6.9 mmol/L and IGT as 2-h plasma glucose of 7.8–11.0 mmol/L. HUA was defined as serum uric acid levels>420μmol/l (>7 mg/dL) in men according to the American College of Rheumatology (1977) (15).

### Genotype analysis

From all study participants, blood samples were taken and biochemical parameters were measured using a Hitachi 2700--310 automatic biochemical analyzer. Genomic DNA was isolated from peripheral blood leukocytes using Relax Gene Blood DNA System (Qiagen Biotech Co., Ltd., Beijing, China) according to the manufacturer’s instructions. A segment of intron 7 of the SLC2A9 gene was amplified by polymerase chain reaction (PCR). The primers of rs734553 were as follows: forward 5′-GGAGAATCTGGAGCAAGT-3′ and reverse 5′-AAATAGTCCCAAAGAGTG-3′ (GenScript Biotech Co., Ltd., Nanjing, China). PCR reactions were performed at 25μL volume containing 50ng genomic DNA, 1 μL of each primer (10 μM), 12.5μL 2×Taq PCR Master Mix (Qiagen Biotech Co., Ltd., Beijing, China) and 8.5 μl ddH_2_O. The PCR cycling conditions were 94 °C for 5 min, followed by 35 cycles at 94°C for 30 sec, 52 °C for 30 sec, 72 °C for 30 sec and a final extension at 72 °C for 10 min. A 3 ul aliquot of PCR product was digested at 37 °C for 2 h in a 10 μL reaction containing 0.3μL restriction enzyme *Hap* II (New England Biolabs, Inc, Beverly, MA), 1 μL 10×NE Buffer (New England Biolabs, Inc, Beverly, MA) and 5.7 μL sterileddH_2_O at 52 °C for 2 h. The genotypes were identified by electrophoresis on 1.5% agarose gels. Five samples in each genotype were sequenced by Sangon Biotech Co., Ltd. (Shanghai, China).

### Statistical analysis

Statistical analyses were performed using SPSS version 13.0 (Chicago, IL, USA). Continuous variables are expressed as the mean ± standard deviation and were analyzed by one-way analysis of variance. The χ^2^ test was used to compare differences in rates. Logistic regression was used to correct for age differences between groups. Values of *P*<0.05 were considered statistically significant. For all subjects, the Hardy–Weinberg equilibrium of the genotype distribution was tested using the homogeneity χ^2^ test.

## Results

There were no significant differences in creatinine levels between the control subjects and those with T2DM. Cholesterol, creatinine, and urea nitrogen levels were not significantly different between the subjects with T2DM and subjects with HUA (all, *P*>0.05). Cholesterol levels were not significantly different between subjects with T2DM without or with HUA. Furthermore, cholesterol and uric acid levels were not significantly different between subjects with HUA and those with T2DM complicated with HUA (Table 1).

**Table 1:** Demographic characteristics and clinical data

***Variable***	***NC***	***T2DM***	***HUA***	***T2DM complicated with HUA***
**n.**	**128**	**167**	**198**	**115**
Age (yr)	41.92±10.01	58.07±11.82	39.20±9.73	51.24±10.09
Glucose (mmol/L)	5.03±0.57	6.94±1.92^a^	5.05±0.39^c,e^	7.02±1.58^a,g,i^
Triglyceride (mmol/L)	0.89±0.36	1.87±1.09^a^	2.79±0.81^a,f^	3.08±1.03^a,e,i^
Cholesterol (mmol/L)	4.80±0.72	4.95±0.79^a^	5.14±0.89^a^	5.34±1.07^a^
Uric acid (μmol/L)	279.76±59.84	312.45±60.15^b^	469.87±50.59^a,e^	489.08±69.38^a,e^
Creatinine (mmol/L)	89.68±9.58	101.68±20.84	97.59±10.95^d^	120.05±90.62^a,h,j^
Urea nitrogen (mmol/L)	4.96±0.78	5.90±1.62^a^	5.47±0.83^a^	5.96±1.70^a^

NC: normal control subjects; T2DM: type 2 diabetes mellitus; HUA: hyperuricemia;

a P< 0.001,

b P = 0.001,

c P = 0.008, and

d P = 0.027 vs. NC;

e P< 0.001,

f P = 0.012,

g P = 0.009, and

h P = 0.001 vs T2DM;

i P< 0.001 and

j P = 0.014 vs. HUA. Other between-group comparisons were not statistically significant (*P*> 0.05)

**Fig. 1:**
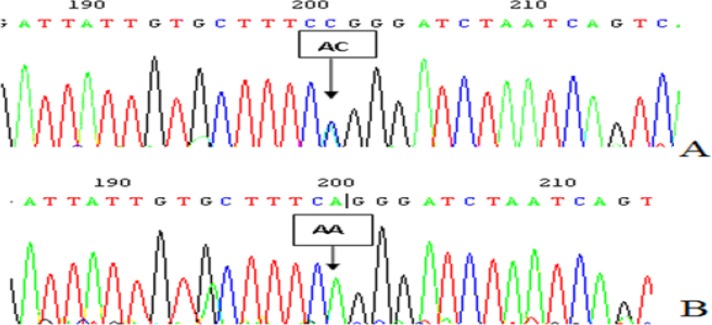
The polymorphism loci rs734553 sequencing figure in 7 introns of SLC2A9 gene A:heterozygoustype (A/C); B: homozygous type (A/A)

The PCR products were the 482 bp nucleotide sequences. Following restriction enzyme digestion of the amplified DNA, the AA genotype was not digested (bands at 482 bp), the AC genotype was heterozygote for digestion with a band (bands at 482, 261 and 221 bp) and the CC genotype was completely missing. The AA and AC genotypes detected by the cleaved amplification polymorphism sequence-tagged sites (PCR-RFLP) were also confirmed by sequencing, respectively (Fig. 1).

The genotype and allele frequencies distributions for SNP rs734553 were in Hardy–Weinberg equilibrium in the individual groups, as the *P*-values of χ^2^tests, were >0.05 in all four groups. The allelic frequency of the rs734553 polymorphism in the control subjects was given in Table 2. None of the study subjects were homozygous for C/C. Accordingly, there were marked differences in the proportions of subjects with the A/A and A/C genotypes in all four groups. Notably, the risk ofT2DM complicated with HUA was significantly higher in people with the C allele than in those with the A allele (95% confidence interval: 0.194–0.502).

**Table 2:** Results of the χ^2^ test of genotype and allele frequencies

***Groups***	***Genotype and genotype frequency***		***Alleles and allelic frequency***	
	**χ^2^**	***P***	**χ^2^**	***P***
A and B	1.463	0.236	1.346	0.26
A and C	4.325	0.045	4.483	0.035
A and D	4.004	0.049	3.943	0.039
B and C	0.053	0.863	0.063	0.742
B and D	0.372	0.638	0.364	0.475
C and D	0.405	0.602	0.401	0.629

A: control subjects; B: subjects with type 2 diabetes mellitus (T2DM); C: subjects with hyperuricemia (HUA); D: subjects with T2DM complicated with HUA

## Discussion

Chronic metabolic diseases have recently gained attention as a major public health problem around the world, especially such as T2DM and HUA. And in 2004 and 2009, we conducted epidemiological investigation of more than 13000. The prevalence of males with HUA has been up to 18.32%, females 8.56% in 2004 (16). In China, the world’s most populous nation has approximately 100 million patients with diabetes(17). T2DM and HUA often occur together. An elevated level of uric acid predicts the development of diabetes (18, 19). And compared with those only suffering from HUA, incidence of gout in those combined with diabetes increases 157.1% (95% CI, 11.0%–495.3%) (20). T2DM and HUA should be paid more attention. However, hereditary factor of the diseases and mutations in genes have been less investigated.

As GLUT9 or (URAT) v1, SLC2A9 situated on chromosome 4p 15.3–16, which encodes a dual transporter for fructose and uric acid is. It has two variants, the long isoform SLC2A9v1 and the short isoform SLC2A9v2 that has been shown to transport urate. Recently, GLUT9 was also considered as a uric acid transporter. GLUT9 was the only member of GLUT family that whose substrate is urate(21). In tandem with URAT1, the renal reabsorption of UA is dependent on two variants, long GLUT9L, and short GLUT9S. What’s more, URAT1 and apical GLUT9S, GLUT9L are the only major urate efflux transporters that mediated urate uptake from the tubular lumen into the cell at the basolateral Membrane (22). SLC2A9 variants influenced uric acid levels in Korean adults (23). Genetic variants of SLC2A9 have close relation with serum uric acid in multiple populations by genome-wide scans (24). Uric acid was identified as a risk factor for the development of type 2 DM in other studies (25). SLC2A9might be associated with type 2 DM and HUA. Although genetic variants of SLC2A9 were strongly associated with serum uric acid levels (26–29); however, the relationship of genetic polymorphisms and DM combined with HUA in Chinese population is rare.

The rs734553 polymorphism is located in intron 7 of the SLC2A9 gene (RefSNP alleles: A/C). SLC2A9 has a relatively conserved amino acid sequence in the seventh and eighth helices located around the central channel of the transport protein (10), the rs734553 polymorphism in intron 7 may alter the polarity of some of these conserved amino acids. Therefore, the polymorphism may affect the transporters affinity for glucose and uric acid, resulting in changes in blood glucose and uric acid levels. The polymorphism is strongly associated with serum uric acid level (30, 31), also demonstrated in our study. The component number of metabolic syndrome can induce the increase of mean serum uric acid level (32, 33), and the prevalence of metabolic syndrome also increases significantly with uric acid level (34, 35). Therefore, both uric acid level and metabolic syndrome are closely linked. As metabolic syndrome is a major contributor to the development of T2DM (36), the rs734553 polymorphism may be the best choice for studying the relation between T2DM and SLC2A9.

We supposed that the SLC2A9 variations might be correlation with HUA complicated with DM. To verify the hypothesis, we analyzed the polymorphisms in SLC2A9 gene rs734553 exon7 and compared the genotypes and frequencies of alleles with the control group. Our study shows that the rs734553 polymorphism is associated with diabetes complicated with HUA (*P*=0.039). The risk of T2DM complicated with HUA is greater in those with the C allele than in those with the A allele (95% confidence interval: 0.194–0.502). The minor C allele of rs734553 may alter the polarity of some of these conserved amino acids, and then influence the prolonged phase of insulin secretion and therefore alter serum glucose levels and increase the risk of developing IFG/IGT(37). To our knowledge, this is the first report on the variant of SLC2A9 in Chinese Han population. As a representative of the SLC2A9 gene variants, rs734553 is showed to be associated with T2DM complicated with HUA patients in China. However, the effect of SLC2A9 variants on serum UA levels was different in different areas, in the population of Framingham and Rotterdam, and also in the island of the Adriatic coast of Croatia, variant c.884G>A was related to elevated concentration of serum uric acid (especially in women), however, this is not the case in African-Americans. While the c.841G>A variant was obviously related to the elevated concentrations of serum uric acid and gout in the population of Han Chinese, Japanese, and Solomon Island, but not in the Eastern and Western Polynesians, and Europeans (38–41). This may be due to different diet and lifestyle can also affect serum UA levels. Besides, SLC2A9 gene transcription regulation may be regulated by the joint actions of multiple polymorphisms, but whether other polymorphisms participate in the process still needs to be further study. Genetic background of the population is also a factor, so, inclusion of people from different areas could decrease regional bias.

In function, GLUT9 expression is up-regulated in the liver and kidney of diabetic mice and influenced insulin secretion in pancreatic β cells (6, 42). Pancreatic β cells control blood glucose concentrations by secreting insulin secretion, characterized by a rapid first phase and a prolonged second phase. In this process, the rapid first phase is mediated by the influx of extra-cellular glucose into β cells, principally via GLUT2. Therefore, GLUT9 is involved in glucose uptake in the prolonged phase of insulin secretion. And because GLUT9 is involved in uric acid excretion as the long isoform (SLC2A9v1) mostly localized at the basolateral membrane of proximal tubule epithelial cells, while the short isoform (SLC2A9v2) is predominantly localized to the apical membrane and has high capacity for transporting urate out of the cell into the proximal tubule (43). The GLUT9 polymorphism, rs734553, is powerful for prediction of CKD progression because it is greatly related to serum uric acid of healthy individuals with normal renal function (14). Genetic variability influence serum uric acid and glucose levels might modify susceptibility to T2DM complicated with HUA. The diagnosis and detection of SLC2A9 gene variants should be caused enough attention in patients of T2DMcomplicated with HUA. To clarify this, functional studies of the SLC2A9 variation need to be conducted in the future.

## Ethical considerations

Ethical issues (Including plagiarism, informed consent, misconduct, data fabrication and/or falsification, double publication and/or submission, redundancy, etc.) have been completely observed by the authors.
